# Cancer Risk in Diabetic Patients Treated with Metformin: A Systematic Review and Meta-analysis

**DOI:** 10.1371/journal.pone.0033411

**Published:** 2012-03-20

**Authors:** Hiroshi Noto, Atsushi Goto, Tetsuro Tsujimoto, Mitsuhiko Noda

**Affiliations:** 1 Department of Diabetes and Metabolic Medicine, Center Hospital, National Center for Global Health and Medicine, Tokyo, Japan; 2 Department of Diabetes Research, Diabetes Research Center, Research Institute, National Center for Global Health and Medicine, Tokyo, Japan; Sapienza University of Rome, Italy

## Abstract

**Background:**

A growing body of evidence has suggested that metformin potentially reduces the risk of cancer. Our objective was to enhance the precision of estimates of the effect of metformin on the risk of any-site and site-specific cancers in patients with diabetes.

**Methods/Principal Findings:**

We performed a search of MEDLINE, EMBASE, ISI Web of Science, Cochrane Library, and ClinicalTrials.gov for pertinent articles published as of October 12, 2011, and included them in a systematic review and meta-analysis. We calculated pooled risk ratios (RRs) for overall cancer mortality and cancer incidence. Of the 21,195 diabetic patients reported in 6 studies (4 cohort studies, 2 RCTs), 991 (4.5%) cases of death from cancer were reported. A total of 11,117 (5.3%) cases of incident cancer at any site were reported among 210,892 patients in 10 studies (2 RCTs, 6 cohort studies, 2 case-control studies). The risks of cancer among metformin users were significantly lower than those among non-metformin users: the pooled RRs (95% confidence interval) were 0.66 (0.49–0.88) for cancer mortality, 0.67 (0.53–0.85) for all-cancer incidence, 0.68 (0.53–0.88) for colorectal cancer (n = 6), 0.20 (0.07–0.59) for hepatocellular cancer (n = 4), 0.67 (0.45–0.99) for lung cancer (n = 3).

**Conclusion/Significance:**

The use of metformin in diabetic patients was associated with significantly lower risks of cancer mortality and incidence. However, this analysis is mainly based on observational studies and our findings underscore the more need for long-term RCTs to confirm this potential benefit for individuals with diabetes.

## Introduction

Hyperinsulinemia and hyperglycemia are thought to promote carcinogenesis in patients with diabetes mellitus. Several meta-analyses have demonstrated that diabetes is associated with increased risks of site-specific cancers of the breast (1.2) [1], endometrium (2.1) [2], bladder (1.2) [3], liver (2.5) [4], colorectum (1.3) [5], and pancreas (1.8–2.1) [6], [7], and also a decreased risk of prostate cancer (0.8–0.9) [8], [9]. The evidence for non-Hodgkin's lymphoma remains inconclusive [10], [11]. Our previous meta-analyses showed that patients with diabetes have an inscreased risk of total cancer (relative risk, 1.1–1.7) [12]–[14], whereas more recent studies did not [15], [16]. Metformin is an insulin sensitizer that is the drug of first choice in the management of type 2 diabetes [17], given its safety profile and lower cost. Metformin reportedly has a potential anti-cancer effect by activating adenosine 5′-mono-phosphate-activated protein kinase (AMPK) in addition to alleviating hyperinsulinemia and hyperglycemia. Although other mechanisms for this risk reduction have been hypothesized, none have been elucidated entirely. Previous meta-analyses have suggested that metformin is associated with a reduced risk of cancer in diabetic subjects [18], [19]. However, those analyses were based solely on a few observational studies and additional reports have been published recently.

In light of the worldwide diabetes epidemic and the higher mortalities in cancer patients with diabetes [20], [21], explorations of effective cancer prevention are of clinical importance for the targeted management of diabetes in daily practice. Moreover, they are crucial in the areas of public health, since a modest increase in the risk of cancer translates into a substantial social burden. These circumstances prompted us to investigate, with greater precision, the preventive effect of metformin on cancer mortality and incidence by scrutinizing pertinent original reports including randomized controlled trials (RCTs), and combining their data in an attempt to obtain meaningful clues for the prevention of cancer in patients with diabetes [13].

## Methods

### Search

Searches of MEDLINE, EMBASE, ISI Web of Science, Cochrane Library, and ClinicalTrials.gov from their inception until October 12, 2011, were performed. Studies evaluating the risks of cancer mortality or incidence among diabetic patients taking metformin, compared with those not taking metformin, were identified using a combination of the following medical subject heading terms: ‘diabetes’, ‘metformin’, ‘cancer’ or ‘neoplasms’, and ‘risk’ or ‘risk factors’. The reference lists of the pertinent articles were also inspected.

### Selection/Study Characteristics

We assessed all the identified RCTs, cohort studies, case-control studies, and cross-sectional studies on the risk of cancer based on original data analyses to determine their eligibility for inclusion in a qualitative analysis. The inclusion criteria in the meta-analysis are as follows: published full-text report in English-language, RCTs with parallel-design of metformin as a treatment of type 2 diabetes at least one year's follow-up period, observational studies of any duration in patients with type 2 diabetes, reporting relative risks, i.e. hazard ratios (HRs), RRs, or odds ratios, adjusted for possible confounders with confidence intervals (CIs). The comparators were defined as any treatment not including metformin.

### Validity assessment

To ascertain the validity of the eligible studies, the quality of each report was appraised in reference to the CONSORT statement [22] and the STROBE statement [23].

### Data abstraction

We reviewed each full-text report to determine its eligibility and extracted and tabulated all the relevant data independently. The extracted data included the characteristics of the subjects (including age, sex, and other treatment), study design, published year, follow-up period, and the methods used for ascertaining the diagnosis of cancer. Study authors were contacted as needed to obtain detailed data. Any disagreement was resolved by a consensus among the investigators.

### Quantitative data synthesis

If more than one study was published for the same cohort, the report containing the most comprehensive information on the population was included to avoid overlapping populations. The reports were summarized both qualitatively and quantitatively. Three articles that did not specify the case numbers were not included in the calculation of the mortality and incidence. If the metformin comparator included more than one treatment, the oral monotherapy groups were included in the analysis because these groups were deemed to be at an equivalent stage of diabetes. If an article provided the relative risks for all cancer and site-specific cancers, the all cancer data were included in the primary qualitative and quantitative analyses and the site-specific data were used in the secondary analyses performed according to cancer site. The risks for site-specific cancers were appraised if three or more qualified reports were identified for a given cancer site. Response to metformin exposure was evaluated by using linear-regression analysis.

In the meta-analysis, each adjusted relative risk was combined and the pooled RRs with the 95% CI was calculated using the random-effects model with inverse-variance weighting. Heterogeneity among the studies was evaluated using I^2^ statistics. The possibility of a publication bias, which can result from the non-publication of small studies with negative findings, was assessed visually using a funnel plot for asymmetry. RevMan (version 5.1) was used for these calculations. A sensitivity analysis was performed by separating the RCTs and the observational cohort / case-control studies and the equality of RRs between RCTs and observational studies were assessed by using *z*-statistic tests. All the procedures were in accordance with the guidelines for the Quality of Reporting of Meta-analyses [24], the meta-analysis of observational studies in epidemiology [25] and the PRISMA statement [26].

## Results

### Search Results

**Figure 1:**
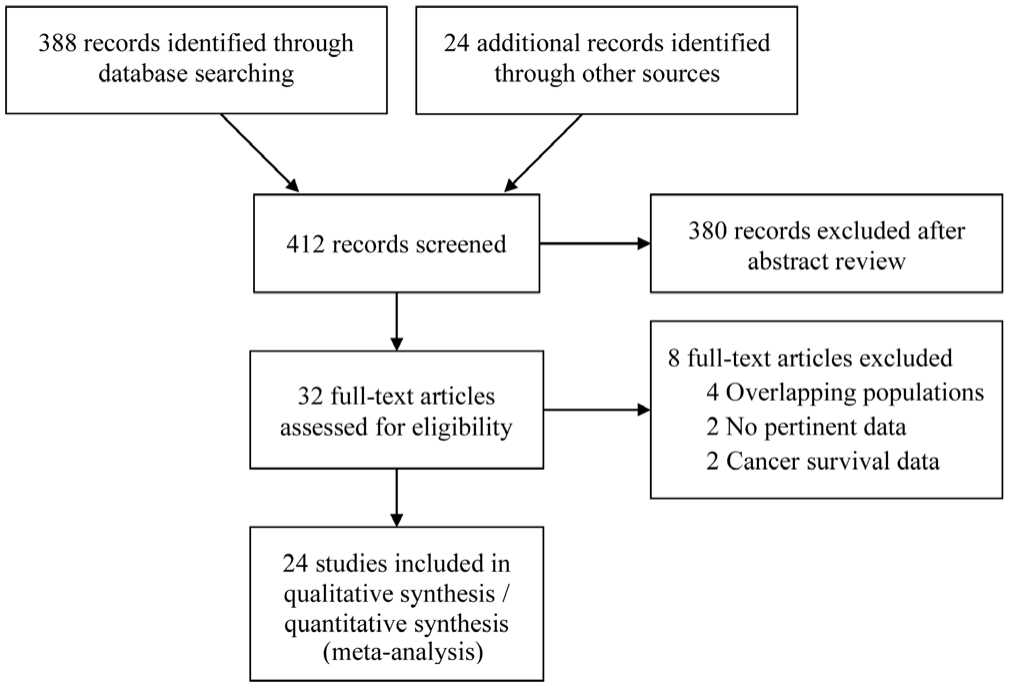
Flow diagram of study selection.

A total of 412 articles were identified during our search; of these, 32 were assessed with respect to their eligibility for inclusion in our review, which was aimed at determining the influence of metformin on cancer mortality and incidence in patients with diabetes (**Fig. 1**). Four articles [27]–[30] were excluded from the systematic review because of population overlapping and four other reports were excluded because they investigated the overall survival rate [31], [32], cancer incidence exclusively in patients with hepatitis C [33], and biochemical recurrence [34]. Out of these 32 articles, a total of 24 (11 observational cohort studies [35]–[45], 3 randomized controlled trials [46]–[49], and 10 case-control studies [29], [50]–[58]) were included in the systematic review and meta-analysis. The UK Prospective Diabetes Study (UKPDS) 34 [49] involved two independent investigational trials (metformin vs. conventional therapy and sulfonylurea vs. sulfonylurea plus metformin), and these trials were included in the meta-analysis as two separate data.

**Table S1** shows the characteristics of each included study according to the study design. The 24 selected articles included in the systematic review were moderately heterogeneous in terms of population demographics, study design, and the assessment of confounding factors. The diabetes sample size in these studies ranged from 361 to 998,947 patients. Of the 21,195 diabetic patients in 6 studies, 991 (4.5%) cases of cancer death were reported. A total of 11,117 (5.3%) cases of incident cancer at any site were reported among 210,892 patients in 10 studies. Major confounding factors such as cigarette smoking, alcohol intake, and hyperglycemia were not reported in several studies.

The risk of bias and the adjustment factors among the studies are summarized in **Table S2**. Diabetes was diagnosed using blood tests (n = 8), prescription databases (n = 6), medical records (n = 4), self-reports (n = 3), and health insurance database (n = 4). All the diagnoses of cancer were confirmed using valid records or registries. All the studies, except for the RCTs, adjusted the estimates for potential confounding factors. The analysis of dose-response was performed in 3 studies [38]–[40]. Some studies excluded the data for metformin exposure less than 1 year [50], [52] or 2 years [58] to minimize bias. The effect on the total cancer risk over the follow-up period was inspected in 3 studies [40], [55], [58]. Direct comparison of the effect between metformin and other specific medications were reported in 2 RCTs [46]–[48].

### Qualitative Summary

The majority of the studies included were methodologically fair in quality. Among 10 case-control studies, six were nested ones [50]–[52], [55], [56], [58]. All the four cohort studies [35], [38], [40], [41] on cancer mortality revealed a significant decrease (range, 23%–75%), and the two RCTs showed no significant effect of metformin [49]. There was no study that directly compared the risk associated with metformin vs other medications or analyzed the correlation between the follow-up length and the effect of metformin on cancer mortality. The overall correlation of the follow-up period with the mortality was nonsignificant (r = −0.04, p = 0.9). One study revealed that the HR (95% CI) for cancer mortality with every increase of 1 g metformin was 0.58 (0.36–0.93) [38].

Five studies (3 cohort studies [36], [39], [40] and 2 case-control studies [55], [56]) reported a significant decrease (range, 26%–88%), the two RCTs showed no significant effect of association [46]–[48] and none demonstrated a statistically significant increase in the risk of all-cancer incidence among metformin users. The cancer risk for metformin users was not significantly different from that for rosiglitazone or sulfonylurea users in RCTs [46]–[48]. One cohort study showed a trend for metformin users to have a higher risk of cancer in the first 2 years of follow-up. The beneficial effect of metformin on the risk of total cancer incidence was exposure-dependent in 2 case-control studies [55], [56]. The overall correlation of the follow-up period with the incidence was nonsignificant (r = −0.32, p = 0.4). One study reported that its effect on cancer incidence was dose-dependent (p for trend <0.05) [39] suggesting that the minimal effective dose can be 500 mg /day, while the other showed no significant differences among doses [40].

Among the studies evaluating the risks of site-specific incident cancers in patients with diabetes who were taking metformin, more than two studies (including subgroup analyses) recognized significantly reduced risks for cancers of the pancreas [36], [39], [54], colorectum [36], [39], [40], and liver [29], [39], [53], and none showed a significantly increased risk of a site-specific cancer. All these risk decrements were moderate (RR range, 0.06–0.60). Of note, no significant increases or decreases in the risk of cancers of the breast, prostate or stomach were reported, except for a significant decrease in the risk of prostate cancer in one report [42] and breast cancer in another [52]. The number of studies examining other cancer sites was two or fewer, and these studies were not reviewed in the present analysis.

### Quantitative Summary (Meta-analysis)

**Figure 2:**
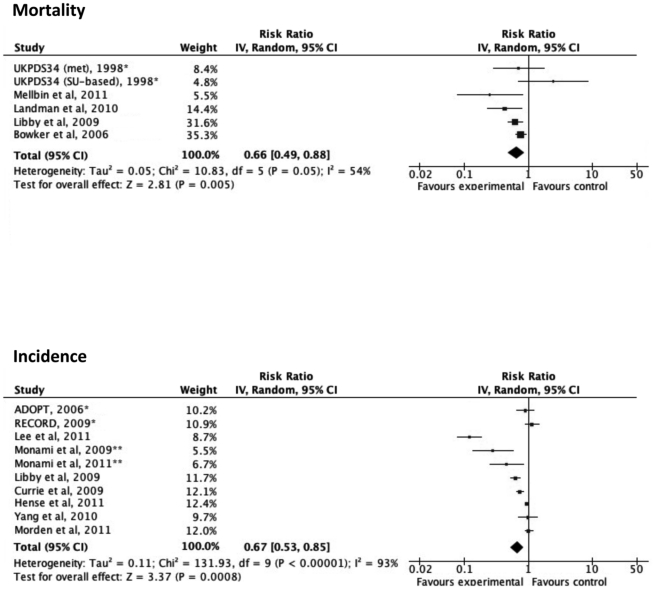
Adjusted risk ratios for all-cancer mortality and incidence among subjects with diabetes taking metformin. Boxes, estimated risk ratios (RRs); bars, 95% confidence intervals (CIs). Diamonds, random-effects model RRs; width of diamonds; pooled CIs. The size of each box is proportional to the weight of each study in the meta-analysis. *, randomized controlled trials; **, case-control studies; IV, inverse-variance.

**Figure 3:**
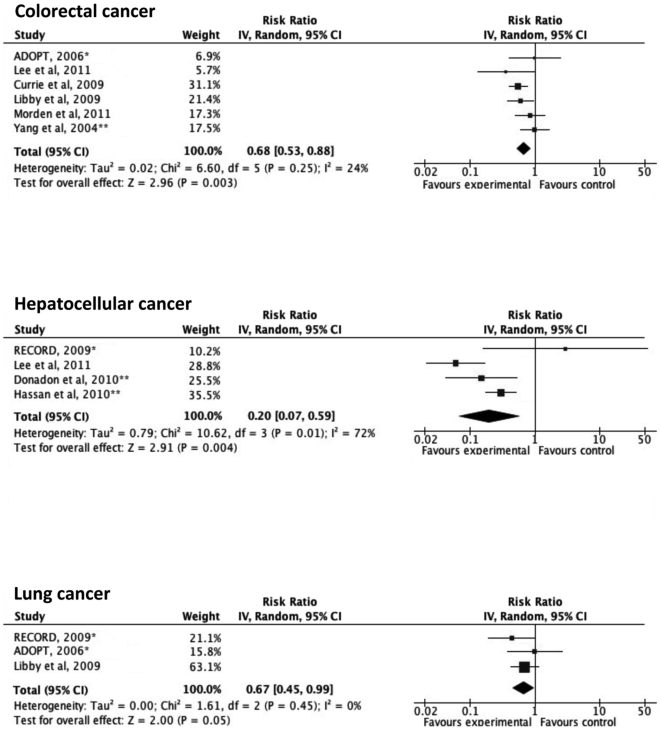
Adjusted risk ratios for site-specific cancer incidence among subjects with diabetes taking metformin. Boxes, estimated risk ratios (RRs); bars, 95% confidence intervals (CIs). Diamonds, random-effects model RRs; width of diamonds; pooled CIs. The size of each box is proportional to the weight of each study in the meta-analysis. *, randomized controlled trials; **, case-control studies; IV, inverse-variance.

**Figure 4:**
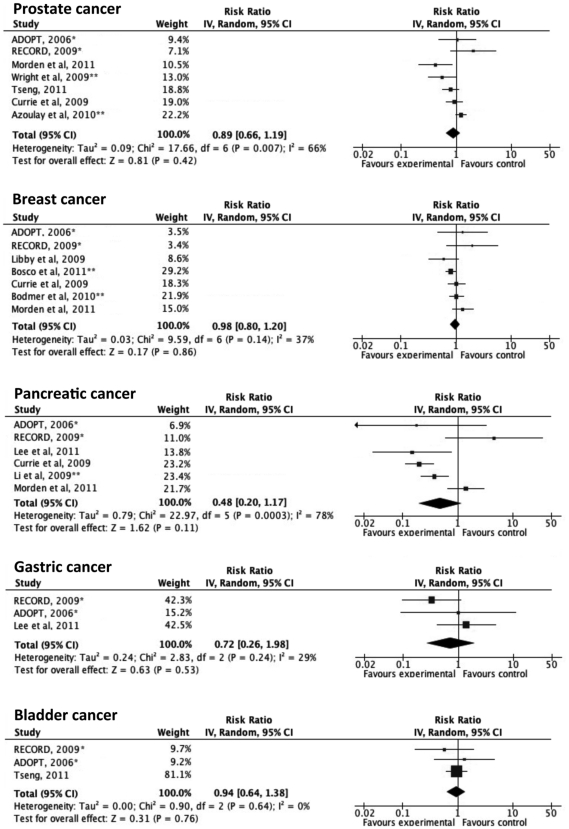
Adjusted risk ratios for other site-specific cancer incidence among subjects with diabetes taking metformin. Boxes, estimated risk ratios (RRs); bars, 95% confidence intervals (CIs). Diamonds, random-effects model RRs; width of diamonds; pooled CIs. The size of each box is proportional to the weight of each study in the meta-analysis. *, randomized controlled trials; **, case-control studies; IV, inverse-variance.

Based on the quality appraisal in our systematic review, a total of 24 articles that provided sufficient information were included in the meta-analysis (**Fig. 1**). **Fig. 2** illustrates the significantly decreased risks of all-cancer mortality and incidence in metformin-users, compared with non-metformin users. In a sensitivity analysis, the pooled estimate (95% CI) for all-cancer mortality among the observational cohort studies was 0.62 (0.46–0.82), I^2^ = 56%, p = 0.08 and the estimate among the RCTs was 1.22 (0.36–4.11), I^2^ = 60%, p = 0.12. The difference in the RRs between the observational studies and the RCTs was not statistically significant (p = 0.35). The pooled RR (95% CI) for all-cancer incidence among the observational cohort studies was 0.66 (0.49–0.88), I^2^ = 96%, p<0.00001, the pooled RR among the case-control studies was 0.38 (0.23–0.61), I^2^ = 3%, p = 0.31 and the estimate among the RCTs was 1.03 (0.82–1.31), I^2^ = 30%, p = 0.23. The difference in the RRs between the observational studies and the RCTs was statistically significant (p = 0.019). As summarized in **Fig. 3** **and** **Fig. 4**, the incident cancer risks were also significantly decreased for cancers of the colorectum, liver and lung. The RRs of prostate cancer, breast cancer, pancreatic cancer and gastric cancer were not statistically significant. Significant heterogeneity was observed in the majority of these analyses. No apparent publication bias was apparent, as assessed using a funnel plot (**Fig. S1**).

## Discussion

Our systematic review and meta-analyses of worldwide reports demonstrated that metformin is associated with a substantially lower risk of all-cancer mortality and incidence, compared with other treatments for diabetes. They also showed that metformin significantly reduced the risks of cancers of the colorectum, liver and lung. These findings support the hypothesis that metformin potentially has an anti-cancer effect. In light of the fact that cancer is the second and diabetes the twelfth leading cause of death worldwide [59] and that the number of people with diabetes is rapidly increasing, our findings have substantial clinical and public implications on a global scale and point to the need for the further investigation of the anti-cancer mechanism of metformin and for long-term RCTs to confirm this clinical benefit.

The strength of our present study is that the analysis was mainly based on large population-based data originating from multiple nations and was performed with a high level of precision. Compared with recently published studies [18], [19], our updated study is novel in that data from RCTs were incorporated and cancer risks for substantially more sites were analyzed. Although the significantly decreased pooled RRs for all-cancer mortality / incidence and cancer at most sites were robust, the results of the component studies were statistically heterogeneous. Of note, all the individual and pooled results of the RCTs were neutral. It seems that each follow-up period in these RCTs is similar to many others in the observational studies and they have power enough to detect the differences in cancer risk. In the analysis of cancer mortality, there was no significant difference in RR between the RCTs and the observational studies. For cancer incidence, on the other hand, the overall RR was significantly reduced but the difference was statistically significant. This discordance may imply that the apparent anti-cancer effect of metformin in observational studies was affected by confounding biases and thus more RCTs are awaited to clarify the effect of metformin on cancer incidence. The large I^2^ values indicated that the range of the plausible risk estimates was wide but no evidence in our analysis suggested that metformin may increase the risk of cancer. These findings may reflect the different mechanisms of cancer prevention at different sites and / or different epidemiological characteristics among the diverse populations included in our study.

Evidence has been accumulating to suggest that diabetic patients have a higher risk of cancer than non-diabetic people [12], [13]. While the mechanisms are yet to be investigated, insulin resistance with secondary hyperinsulinemia is the most frequently proposed hypothesis, as insulin may have a possible mitogenic effect via its binding to the insulin-like growth factor-1 receptor [60]–[70]. In addition, hyperglycemia itself may promote carcinogenesis directly [71], [72] or indirectly by increasing oxidative stress [73]–[79]. However, these speculations are derived from retrospective observational studies and may not necessarily demonstrate causality because of possible biases and confounders, such as co-existing obesity and age [15], [80], [81]. In fact, more recent studies demonstrated no or minimal increments in cancer risk [15], [16] and the data from insulin-treated patients are inconclusive [82]. Of interest, diabetes reportedly protects against the development of prostate cancer [8], [9], since it is testosterone-dependent and testosterone deficiency is common among men with diabetes secondary to low levels of sex hormone-binding globulin (SHBG) and partially because of insulin resistance [83]–[85]. Low SHBG levels may facilitate the conversion of testosterone to estradiol, which in turn may result in an increased risk of hormone-dependent breast cancer.

Several mechanisms for the anti-cancer effect of metformin have been postulated, and several prospective clinical trials to evaluate its safety and efficacy are ongoing [82], [86]. Indirect pathways include the prevention of weight gain and the amelioration of hyperinsulinemia, both of which may promote carcinogenesis. In addition, metformin activates AMPK through LKB-1, a tumor suppressor protein kinase. AMPK inhibits protein synthesis and gluconeogenesis during cellular stress and inhibits mammalian target of rapamycin (mTOR), a downstream effector of growth factor signaling, which is frequently activated in malignant cells. In human breast cancer cells, it reduces HER-2 protein expression by inhibiting mTOR. Metformin also induces cell cycle arrest and apoptosis and reduces growth factor signaling. Supporting the idea of these direct effects, metformin reportedly potentiated the effect of neoadjuvant chemotherapy in early-stage breast cancer [87], decreased the risk of colorectal cancer in a small randomized trial involving non-diabetic subjects [88], and was associated with a decreased cancer risk while another insulin-sensitizer, thiazolidinedione, were not [18], [54], [89], [90].

Our research revealed that metformin use is associated with reduced mortality and incidence of cancer at any site, supporting the general applicability of the proposed anti-cancer mechanisms. The anti-cancer effect of metformin may also be applicable to diabetic Asians, who are generally lean and insulinopenic [12], given the fact that they have a higher cancer risk than non-diabetic Asians [12]–[14] and the data for Asians [39] were in line with the results of our meta-analyses. On the other hand, the magnitude of the risk reduction varies among site-specific cancers. This variance in efficacy may result from differences in carcinogenesis at certain sites. For instance, elevated levels of insulin and glucose may exert an important influence in the development or growth of epithelial malignant tumors of the colon [91]–[93], pancreas [94], [95], and breast [96], and metformin may prevent incident colon cancer in non-diabetic subjects [88]. An animal study suggested that metformin prevented smoking-related lung cancer in mice, probably by inducing some hormone from the liver [97]. With regard to sex hormone-dependent cancers, the effect of metformin on the development of prostate cancer and breast cancer in our analysis was neutral. Metformin improves insulin sensitivity, thereby possibly raising the testosterone level. This may have promoted prostate cancer development and may have diluted the beneficial effect of metformin. In fact, one cohort study reported no benefit of metformin in terms of the biochemical recurrence rate after radical prostatectomy in diabetic patients [34]. The nonsignificant pooled RR for breast cancer may have resulted from the diversity in confounder adjustments and follow-up periods: some analyses were not fully adjusted for risk factors, including the menopause status, and one study suggested that only long-term exposure to metformin reduced the risk of breast cancer [51]. The fact that one preliminary study suggested a promising effect of metformin on pathologic complete responses to neoadjuvant chemotherapy in diabetic patients with breast cancer [87] may point to the possibility that metformin simply augmented the efficacy of chemotherapy for breast cancer [18], [86]. Further detailed studies to analyze the interaction between carcinogenesis and the action of metformin, and to evaluate its effect for nondiabetic people are eagerly awaited.

### Limitations

Our analysis should be interpreted in the context of the following limitations. First, the relation may not necessarily be causal, particularly in the observational studies [80], because of possible confounding factors and biases that may not have been fully adjusted for in this study: some risk factors such as cigarette smoking, alcohol intake, and hyperglycemia were not specified in several studies, which may have rendered the results less valid. Few studies demonstrated the dose-response to support biological plausibility. Confounding by treatment indication [98], which may have been minimizes by using propensity-score matching analysis, might overestimate the effect of metformin: the presence of such pre-existing conditions as older age and liver disease precludes metformin usages and thus, metformin users may be generally younger and at lower risk of cancer than in those in comparator groups. Only a few observational studies analyzed the effects over time and thus protopathic bias (i.e. early cancer leading to unstable diabetes and hyperglycemia, with patients switching diabetes treatment) [15] may remain moderate. In fact, the individual and pooled estimates from the RCTs were all neutral; the estimates comparing with other medication were neutral, as well. For all these limitations, however, observational studies provide the good available evidence regarding potential treatment effects / harms and the overall pooled estimates were robust. Moreover, evidence has been accumulating to support causality, both clinically and biochemically, as discussed earlier. Secondly, it is also important to realize that the populations of the studies were heterogeneous, most likely because of the diversity of the study designs and ethnicities, and that the sensitivity of each site-specific cancer to metformin may vary. Lack of the standardized treatment protocol in the descriptive studies might explain the observed associations: the possibility that other diabetes treatments may increase the risk of cancer may have resulted in an overestimation of the effect of metformin. Lack of the standardized diagnostic procedures for cancer may have caused detection bias in some cases. Even with these limitations, our analysis supports oncogenic safety of metformin and it should provide physicians with an additional incentive to pay integrated clinical attention and elucidate the complex interactions between diabetes treatment and cancer.

### Conclusions

Our meta-analysis favors the oncogenic benefit of metformin for diabetic patients. However, observational studies were moderately heterogeneous and biased, and RCTs did not show a significant effect. Our findings underscore the need for long-term randomized prospective studies to confirm this potential benefit.

## Supporting Information

Figure S1 **Funnel plot of the included studies.**
(TIFF)

Table S1 **Study characteristics.**
(DOC)

Table S2 **Quality assessments of the included studies.**
(DOC)

Checklist SI **PRISMA Checklist.**
(PDF)
