# Supplementary figures and images for: Cancer Risk in Diabetic Patients Treated with Metformin: A Systematic Review and Meta-analysis
Source: PLoS One. 2012 Mar 20;7(3):e33411. doi: 10.1371/journal.pone.0033411 (PMC3308971; doi:10.1371/journal.pone.0033411)

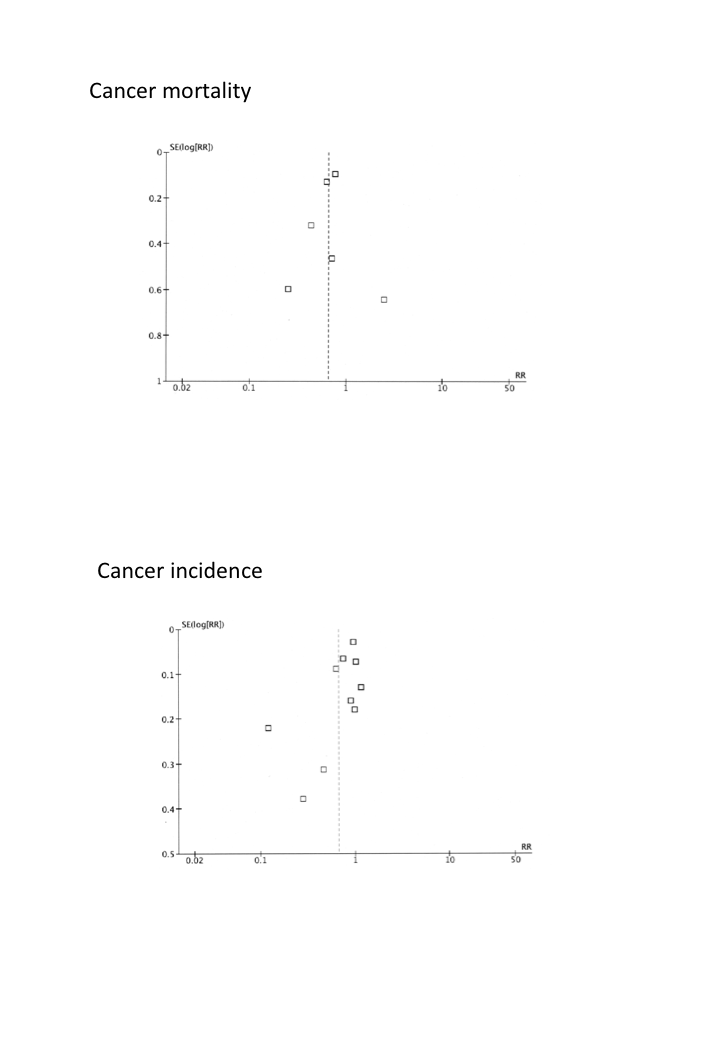

Supplement: Figure S1 — Funnel plot of the included studies. (TIFF) [file pone.0033411.s001.tif]
