# Supplementary material for: Cancer Risk in Diabetic Patients Treated with Metformin: A Systematic Review and Meta-analysis
Source: PLoS One. 2012 Mar 20;7(3):e33411. doi: 10.1371/journal.pone.0033411 (PMC3308971; doi:10.1371/journal.pone.0033411)
Supplement: Table S1 — Study characteristics. (DOC) [file pone.0033411.s002.doc]

Table S1. Study characteristics.

| **Source** | **Country** | **Subject source** | **Follow-up, yr** | **N (men, %)** | **Age, yr** | **Current smoker (%)** | **Alcohol intake none / social / regular / dependent** | **Mean HbA1c(%) metformin users vs comparators** | **Cancer site** | **Cancer case, n** |
| --- | --- | --- | --- | --- | --- | --- | --- | --- | --- | --- |
| **Cohort studies** |  |  |  |  |  |  |  |  |  |  |
| **Observational studies** |  |  |  |  |  |  |  |  |  |  |
| Bowker et al, 2006 | Canada | Population-based | Mean 5.4 | 10309 (55) | Mean 63 | NR | NR | NR | Any site | 407 (mortality) |
| Currie et al, 2009 | UK | Clinic-based | Mean 2.4 | 62809 (54) | Mean 62 | 66 (ever) | NR | 8.4 | Any site | 2106 (incidence) |
|  |  |  |  |  |  |  |  |  |  | Breast: 305 |
|  |  |  |  |  |  |  |  |  |  | Colorectum: 292 |
|  |  |  |  |  |  |  |  |  |  | Pancreas: 89 |
|  |  |  |  |  |  |  |  |  |  | Prostate: 301 |
| Hense et al, 2011 | Germany | Population-based | Median 3.5 | 26742 (47) | Mean 64 | 17 | NR | NR | Any site | 1364 (incidence) |
| Landman et al, 2010 | Netherland | Clinic-based | Median 9.6 | 1353 (42) | Mean 68 | 19 | NR | 7.7 / 7.4 | Any site | 122 (mortality) |
| Lee et al, 2011 | Taiwan | Population-based | Mean 3.8 | 15717 (55) | 20 or older | NR | NR | NR | Any site | 339 (incidence) |
|  |  |  |  |  |  |  |  |  |  | Liver: 73 |
|  |  |  |  |  |  |  |  |  |  | Colorectum: 56 |
|  |  |  |  |  |  |  |  |  |  | Pancreas: 28 |
|  |  |  |  |  |  |  |  |  |  | Stomach: 34 |
| Libby et al, 2009 | UK | Population-based | Maximum 10 | 8170 (54) | Range 35-100 | 14 | NR | 7.9 / 7.2 | Any site | 771 (incidence) |
|  |  |  |  |  |  |  |  |  |  | Breast: 65 |
|  |  |  |  |  |  |  |  |  |  | Bowel: 116 |
|  |  |  |  |  |  |  |  |  |  | Lung: 93 |
|  |  |  |  |  |  |  |  |  |  | 371 (mortality) |
| Mellbin et al, 2011 | European countries | Research center-based | Median 4.1 | 1073 (67) | Mean 68 | 24 | NR | 7.7 (total) | Any site | 37 (mortality) |
| Morden et al, 2011 | US | Population-based | Median 1.9 | 81681 (31) | Mean 77 | 27 | NR | NR | Any site | 5466 (incidence) |
|  |  |  |  |  |  |  |  |  |  | Breast: 553 |
|  |  |  |  |  |  |  |  |  |  | Colon: 428 |
|  |  |  |  |  |  |  |  |  |  | Pancreas: 204 |
|  |  |  |  |  |  |  |  |  |  | Prostate: 427 |
| Tseng, 2011 | Taiwan | Population-based | 3 | 52133 (100) | All ages | NR | NR | NR | Prostate | 362 (incidence) |
| Tseng, 2011 | Taiwan | Population-based | 3 | 998947 (50) | All ages | NR | NR | NR | Bladder | 589 (incidence) |
| Yang et al, 2010 | Hong Kong | Hospital-based | Median 4.9 | 6103 (46) | Mean 57 | 15 | 7.3 (current drinker) | 7.2 (total) | Any site | 271 (incidence) |
| **RCTs** |  |  |  |  |  |  |  |  |  |  |
| ADOPT, 2006 | US, Canada, European countries | Research center-based | Median 4.0 | 4351 (58) | Mean 57 | NR | NR | 7.4 / 7.4 | Any site | 180 (incidence) |
|  |  |  |  |  |  |  |  |  |  | Breast: 15 |
|  |  |  |  |  |  |  |  |  |  | Colorectum: 21 |
|  |  |  |  |  |  |  |  |  |  | Stomach: 3 |
|  |  |  |  |  |  |  |  |  |  | Pancreas: 5 |
|  |  |  |  |  |  |  |  |  |  | Prostate: 29 |
|  |  |  |  |  |  |  |  |  |  | Lung: 18 |
| RECORD, 2009 | European countries, Australasia | Research center-based | Mean 5.5 | 4447 (52) | Mean 58 | 16 | NR | 7.9 / 8.0 | Any site | 313 (incidence) |
|  |  |  |  |  |  |  |  |  |  | Breast: 28 |
|  |  |  |  |  |  |  |  |  |  | Stomach: 10 |
|  |  |  |  |  |  |  |  |  |  | Liver: 4 |
|  |  |  |  |  |  |  |  |  |  | Pancreas: 15 |
|  |  |  |  |  |  |  |  |  |  | Prostate: 36 |
|  |  |  |  |  |  |  |  |  |  | Lung: 21 |
| UKPDS 34 (monotherapy), 1998 | UK | Research center-based | Median 10.7 | 753 (46) | Mean 53 | 25 | 29 / 57 / 14 / 1 | 7.3 / 7.1 | Any site | 34 (mortality) |
| UKPDS 34 (SU-based), 1998 | UK | Research center-based | Median 6.6 | 537 (60) | Mean 59 | 27 | 35 / 48 / 17 / 0.8 | 7.5 / 7.6 | Any site | 20 (mortality) |
| **Case-control studies** |  |  |  |  |  |  |  |  |  |  |
| Azoulay et al, 2010 | UK | Clinic-based | Mean 4.7 (nested) | 8098 (100) | Mean 74 | 64 (ever) | NR / NR / NR / 2 | 7.1 | Prostate | 739 (incidence) |
| Bodmer et al, 2010 | UK | Clinic-based, insulin-treated | Mean 10 (nested) | 1458 (0) | Mean 68 | 15 | NR | Mean not calculated | Breast | 305 (incidence) |
| Bosco et al, 2011 | Denmark | Population-based | Maximum 20 (nested) | 4323 (0) | 50 or older | NR | NR | NR | Breast | 393 (incidence) |
| Donadon et al, 2010 | Italy | Hospital-based |  | 2306 (80) | Mean 69 | NR | NR / NR / NR / 16 | NR | Liver | 610 (incidence) |
| Hassan et al, 2010 | USA | Hospital-based |  | 1524 (61) | Mean 62 | 54 | 10 (>60ml/day) | NR | Liver | 420 (incidence) |
| Li et al, 2009 | USA | Hospital-based |  | 361 (66) | All ages | NR | NR | NR | Pancreas | 255 (incidence) |
| Monami et al, 2009 | Italy | Hospital-based | Mean 6.5 (nested) | 390 (59) | Mean 69 | 19 | NR / NR / NR / 15 | 7.7 | Any site | 195 (incidence) |
| Monami et al, 2011 | Italy | Hospital-based | Median 6.3 (nested) | 482 (52) | Mean 68 | 19 | NR | NR | Any site | 112 (incidence) |
| Wright et al, 2009 | USA | Population-based, Caucasians |  | 1687 (100) | Range 35-74 | NR | NR | NR | Prostate | 843 (incidence) |
| Yang et al, 2004 | UK | Clinic-based | Mean 5.6 (nested) | 1320 (51) | Mean 75 | 10 | NR | NR | Colorectum | 125 (incidence) |

RCT, randomized controlled trial.
