# Supplementary material for: Cancer Risk in Diabetic Patients Treated with Metformin: A Systematic Review and Meta-analysis
Source: PLoS One. 2012 Mar 20;7(3):e33411. doi: 10.1371/journal.pone.0033411 (PMC3308971; doi:10.1371/journal.pone.0033411)
Supplement: Table S2 — Quality assessments of the included studies. (DOC) [file pone.0033411.s003.doc]

**Table S2.** Quality assessments of the included studies.

| **Source** | **Cancer ascertainment** | **Comparator** | **Adjustment factors** |
| --- | --- | --- | --- |
| **Cohort studies** |  |  |  |
| **Observational studies** |  |  |  |
| Bowker et al, 2006 | Population registries | Sulfonylurea | Age, sex, insulin, comorbidity |
| Currie et al, 2009 | Medical records | Sulfonylurea*, metformin + sulfonylurea, insulin | Age, sex, smoking, cancer history, HbA1c, diabetes duration, weight |
| Hense et al, 2011 | Population registries | Other anti-diabetic medications | Age, sex,diabetes duration, BMI, medications |
| Landman et al, 2010 | Medical records | No metformin use | Smoking, age, sex, diabetes duration, HbA1c, serum creatinine, BMI, blood pressure, total cholesterol-to-HDL-C ratio, albuminuria, insulin, sulfonylurea, macrovascular complications |
| Lee et al, 2011 | Health insurance database | No metformin use | Age, gender, other oral antihyperglycemic medication, comorbidity |
| Libby et al, 2009 | Population registries | No metformin use | Age, sex, smoking, deprivation, BMI, HbA1c, insulin, sulfonylurea |
| Mellbin et al, 2011 | Medical records, death certificates | No metformin use | Sex, age, smoking habits, previous myocardial infarction/congestive heart failure, creatinine, percutaneous transluminal coronary angioplasty / coronary artery bypass grafting during hospitalization, blood glucose |
| Morden et al, 2011 | Health insurance database | No metformin use | Age, race/ethnicity, diabetes complications, obesity, oral estrogen use, poverty indicator, comorbidities, tobacco exposure |
| Tseng, 2011 | Health insurance database | No metformin use | Age, diabetes duration, comorbidities, medications, living region, occupation |
| Tseng, 2011 | Health insurance database | No metformin use | Age, sex, diabetes, nephropathy, urinary tract diseases, comorbidities, medications, living region, occupation |
| Yang et al, 2010 | Medical records | No metformin use | Age, sex, body mass index, smoking status, alcohol use status, HbA1c, systolic blood pressure, LDL-C related risk, spline functions of HDL-C and triglyceride for cancer, statins, RAS inhibitor, insulin |
| **RCTs** |  |  |  |
| ADOPT, 2006 | Event records | Rosiglitazone, sulfonylurea | None |
| RECORD, 2009 | Event records | No metformin use | None |
| UKPDS 34 (monotherapy), 1998 | Event records | Conventional therapy | None |
| UKPDS 34 (SU-based), 1998 | Event records | No metformin use | None |
| **Case-control studies** |  |  |  |
| Azoulay et al, 2010 | Medical records | No metformin use | HbA1c, excessive alcohol, obesity, smoking, lower urinary tract symptoms, previous cancer, NSAIDs, antihypertensive drugs, statins, other antidiabetic agents |
| Bodmer et al, 2010 | Medical records | No metformin use | Age, calendar time, general practice, oral antidiabetes drugs, insulin, estrogens, smoking BMI, diabetes duration, HbA1c |
| Bosco et al, 2011 | Medical records | No metformin use | Age, diabetes complications, obesity, postmenopausal hormone use, parity |
| Donadon et al, 2010 | Medical records | No metformin use | Age, sex, BMI, alcohol abuse, hepatitis B virus, hepatitis C virus, ALT, triglyceride, cholesterol, antidiabetes drugs, diabetes duration |
| Hassan et al, 2010 | Medical records | No metformin use | Age, sex, race, educational level, cigarette smoking, alcohol drinking, hepatitis C virus, hepatitis B virus, family history of cancer |
| Li et al, 2009 | Medical records | No metformin use | Age, sex, race, cigarette smoking, alcohol drinking, BMI, family history of cancer, diabetes duration, insulin |
| Monami et al, 2009 | Medical records | No metformin use | Age, sex, diabetes duration, BMI, HbA1c, comorbidity, smoking, alcohol abuse, concomitant therapies |
| Monami et al, 2011 | Medical records | No metformin use | Age, sex, BMI, comorbidity, glargine, insulin doses |
| Wright et al, 2009 | Population registries | No metformin use | Age, other diabetic treatments, aspirin and NSAID usage, BMI, PSA tests in preceding 5 years, family history of prostate cancer |
| Yang et al, 2004 | Medical records | No metformin use | Sex, history of cholecystectomy, smoking, duration of type 2 diabetes mellitus, BMI, sulfonylurea, NSAID/aspirin |

* Included in meta-analysis. RCT, randomized controlled trial; BMI, body mass index; RAS, renin-aldosterone system; PSA, prostate-specific antigen; NSAID, nonsteroidal anti-inflammatory drug.
